# The Power of Music to Prevent and Control Emerging Infectious Diseases

**DOI:** 10.3389/fmed.2021.756152

**Published:** 2021-11-25

**Authors:** Julio A. Benavides, Cristina Caparrós, Ramiro Monã da Silva, Tiziana Lembo, Philip Tem Dia, Katie Hampson, Feliciano Dos Santos

**Affiliations:** ^1^Departamento de Ecología y Biodiversidad, Facultad de Ciencias de la Vida, Universidad Andrés Bello, Santiago, Chile; ^2^Centro de Investigación para la Sustentabilidad, Facultad de Ciencias de la Vida, Centro de Investigación Para la Sustentabilidad, Universidad Andrés Bello, Santiago, Chile; ^3^Department of Veterinary Hygiene and Public Health, São Paulo State University, Botucatu, Brazil; ^4^Institute of Biodiversity, Animal Health and Comparative Medicine, College of Medical, Veterinary and Life Sciences, University of Glasgow, Glasgow, United Kingdom; ^5^Boyd Orr Centre for Population and Ecosystem Health, Institute of Biodiversity, Animal Health and Comparative Medicine, College of Medical, Veterinary and Life Sciences, University of Glasgow, Glasgow, United Kingdom; ^6^Flora and Fauna International, Zor Zor, Liberia; ^7^Fundación Estamos, Lichinga, Mozambique

**Keywords:** art, prevention, Ebola, one health, COVID-19, pandemic, health intervention, HIV

## Abstract

Music is a powerful approach to engage communities and disseminate information. Specifically, health campaigns employing music have been used to promote behaviors that can prevent emerging infectious diseases (EIDs). For example, hip hop artists supported campaigns to prevent acquired immunodeficiency syndrome in the 70s in the United States, while Brazilian funk promoted vaccination to mitigate the ongoing COVID-19 pandemic. Similarly, we broadcast musical messages in local languages to increase community awareness and support prevention measures in Guinea and Liberia in response to the recent Ebola outbreak in 2021. Given the potential of music to promote both individual and population-level behavioral changes to prevent transmission, there is a need to consolidate information on music-based health interventions, and on how we can measure their effectiveness. In this perspective, we provide examples of relevant initiatives, discussing challenges and solutions associated with implementing interventions based on our experience with the 2021 Ebola outbreak. We recommend four steps for a successful music-based health intervention including (1) establishing a task force, (2) compose a “catchy” song including critical preventive measures, (3) deliver the song to the target audience, and (4) evaluate the campaign effectiveness. We argue that close interactions between scientists and musicians can produce rapid musical content for disease prevention. We also identify and discuss several methodological frameworks for testing the effectiveness of such interventions. We conclude that support from public health authorities, government media departments, and international agencies, is necessary to deliver wide outreach and long-term sustainability of musical messaging toward effective EID prevention.

## Introduction

Emerging infectious diseases (EIDs) can have catastrophic impacts on global health and the economy (1–3). Emerging infectious diseases typically originate from a rare spillover event from a wildlife host. Noteworthy examples include the 2014 outbreaks of Ebola virus disease in West Africa (4–6) and the current coronavirus disease (COVID-19) pandemic (7–9). Such unpredictable events can spark outbreaks that rapidly spread through human population causing thousands of fatalities and costing billions of dollars (10, 11). It is widely accepted that immediate preventive measures aiming to limit the initial spread of an EID are needed and are more cost-effective than controlling disease once it has escalated into a larger outbreak (12–14). However, the public health sector and the scientific community have had limited success in delivering timely and socially-acceptable interventions to contain EIDs (15, 16). The role of affected communities in contributing to health interventions, and therefore the need to engage them in the initial stages of response to an outbreak, are increasingly recognized (17, 18). The benefits of community engagement in health promotion are multifaceted, from improved practices to more sustainable and context-specific health messaging and policies (6, 19). Consensus has emerged on the need to use more inclusive approaches to deliver public health interventions, with involvement of target populations and beneficiaries critical to the development of locally relevant preventative messaging.

Art-based approaches have often been proposed as an efficient tool for mass delivery of culturally acceptable messages to improve health (20–22). In particular, music has been used by the public sector and non-governmental organizations (NGOs) to promote disease prevention, delivering health information in a format that better resonates with the general public or with specific target groups (e.g., teenagers). Psychological models have been proposed to promote health-protective behaviors (15). Some focus on encouraging individual-level changes of health-related risk perceptions and behaviors [see Tengland et al. (23) for examples on the behavior change and empowering models]. Others operate at the population level, and therefore need to account for social processes, factors enabling community engagement, cultural beliefs, or socioeconomic conditions (15, 24). Within these theoretical frameworks, music-based interventions focusing on EIDs could (a) contribute to increasing awareness on risky and protective behaviors, (b) address cultural factors that might affect uptake of disease prevention or encourage stigmatizing behaviors, and (c) build trust between health professionals and target populations (23–25). In this perspective, we provide examples of how music has been used in infectious disease prevention with an emphasis on EIDs, and propose four critical steps to enhance its potential describing our recent experience during the 2021 Ebola outbreak in Guinea.

## Music-Based Campaigns to Prevent Infectious Diseases

Music-based health interventions have been developed and implemented for several endemic and emerging diseases, including human immunodeficiency virus/acquired immunodeficiency syndrome (HIV/AIDS), Ebola, malaria, and COVID-19 (Table 1). One of the first examples of the use of music in health promotion is hip hop music in prevention campaigns for HIV/AIDS in the 1970s in the United States (36). This genre targets African and Latin American youth, groups who are most impacted by this disease (43). Musical health-related messaging is also widely disseminated online (see Supplementary Table 1 for examples posted on YouTube), although these initiatives are undocumented in published literature. For example, COVID-specific music-based preventive messages have achieved high visibility, including songs by the singer Khac Hung in Vietnam (101 million views) and by the Brazilian funk rapper MC Fioti (13 million views).

**Table 1:** Examples of music interventions to prevent and control endemic and emerging infectious diseases retrieved from PubMed published between 2006 and 2021.

**Disease**	**Ref**.	**Title**	**Country**	**Target population**	**Delivery media**	**Year**
COVID	Kitara and Ikoona (26)	COVID-19 pandemic, Uganda's story	Uganda	General population	Social media, Radio, Television	2020
	Cournoyer Lemaire (27)	Extraordinary times call for extraordinary measures: the use of music to communicate public health recommendations against the spread of COVID-19	Canada	General population	Not defined	2020
	Deng et al. (28)	Global COVID-19 Advertisements: Use of Informational, Transformational and Narrative Advertising Strategies	49 countries (Worldwide)	General population	Web	2020
	Appiah et al. (29)	Promoting COVID-19 vaccination through music and drama-Lessons from early phase of the pandemic	Global scale	General population	Live, Social media, Radio	2021
	Thompson et al. (30)	Communicating Awareness About COVID-19 Through Songs: An Example From Ghana	Ghana	General population	Social media, Radio, Television	2021
	de-Graft Aikins and Akoi-Jackson (31)	“Colonial Virus”: COVID-19, creative arts and public health communication in Ghana	Ghana	General population	Newspaper, Social media	2020
	Thampi et al. (32)	It's in our hands: a rapid, international initiative to translate a hand hygiene song during the COVID-19 pandemic	Global scale	Children	Web	2020
Ebola	Stürmer et al. (33)	Mobilizing the global community to combat Ebola: Psychological effects of the Band Aid 30 campaign	Germany	University students	Web	2016
Hand germs	Younie et al. (34)	Improving young children's handwashing behavior and understanding of germs: The impact of A Germ's Journey educational resources in schools and public spaces	United Kingdom	Children	Live	2020
Helminths	Al-Delaimy et al. (35)	Developing and evaluating health education learning package (HELP) to control soil-transmitted helminth infections among Orang Asli children in Malaysia	Malaysia	Children	Live	2014
HIV	Turner-Musa et al. (36)	Hip-hop to prevent substance use and HIV among African-American youth: a preliminary investigation	United States of America	Urban adolescents	Live	2008
	Lemieux et al. (25)	A music-based HIV prevention intervention for urban adolescents	United States of America	Urban adolescents	Live	2008
	Glenn and Wilson (37)	African American adolescent perceptions of vulnerability and resilience to HIV	United States of America	Urban adolescents	Live	2008
	Diclemente et al. (38)	African-American men's exposure to music videos and their sexual attitudes and risk behavior	United States of America	Adult men	Live	2013
	Harris et al. (39)	Condom social marketing program to prevent HIV/AIDS in post-conflict Liberia	Liberia	Urban adolescents	Live	2011
	Muñoz-Laboy et al. (40)	Condom use and hip hop culture: the case of urban young men in New York City	United States of America	Urban adolescents	Live	2008
	Geary et al. (41)	Does MTV reach an appropriate audience for HIV prevention messages? Evidence from MTV viewership data in Nepal and Brazil	Nepal and Brazil	MTV audience	Television	2006
	Holstad et al. (42)	Focus Group Evaluation of the LIVE Network-An Audio Music Program to Promote ART Adherence Self-Management	United States of America	HIV-infected persons	Television, Radio	2012
	Boutin-Foster et al. (43)	Reducing HIV and AIDS through Prevention (RHAP): a theoretically based approach for teaching HIV prevention to adolescents through an exploration of popular music	United States of America	Urban adolescents	Live	2010
	Bastien (44)	Reflecting and shaping the discourse: the role of music in AIDS communication in Tanzania	Tanzania	Young population	Radio	2009
	Minc et al. (45)	The Jailbreak Health Project–incorporating a unique radio programme for prisoners	Australia	Prisoners	Radio	2007
	Bull et al. (46)	What do young adults expect when they go online? Lessons for development of an STD/HIV and pregnancy prevention website	United States of America	Young population	Web	2007
	Rodriguez et al. (47)	Feasibility and Acceptability of an Adolescent-Friendly Rap Video to Improve Health Literacy Among HIV-Positive Youth in Urban Peru	Peru	Urban adolescents	Live	2021
	Yoshida et al. (48)	Evaluating educational media using traditional folk songs ('lam') in Laos: a health message combined with oral tradition	Laos	General population	Live	2012
	Stadler et al. (49)	Hold on' (Bambelela)! Lyrical interpretations of participation in an HIV prevention clinical trial	South Africa	Young population	Live	2018
Malaria	Paul and Pal (50)	Intervention on malaria awareness among 'Bedia' tribal community in West Bengal, India	India	“Bedia” tribal community	Live	2019
	Peto et al. (51)	Reflections on a Community Engagement Strategy for Mass Antimalarial Drug Administration in Cambodia	Cambodia	Villages population	Live	2018
	Anderson et al. (52)	Using participatory risk analysis to develop a song about malaria for young children in Limpopo Province, South Africa	South Africa	Children	Live	2018
	Manana et al. (53)	“Maskandi experience”: exploring the use of a cultural song for community engagement in preparation for a pilot Sterile Insect Technique release programme for malaria vector control in KwaZulu-Natal Province, South Africa 2019	South Africa	General population	Live	2021
	Eskenazi et al. (54)	A community-based education programme to reduce insecticide exposure from indoor residual spraying in Limpopo, South Africa	South Africa	Villages population	Live	2019
	Panter-Brick et al. (55)	Culturally compelling strategies for behavior change: a social ecology model and case study in malaria prevention	Gambia	Rural areas	Live	2006
	Callery et al. (56)	Engaging ethnic minority communities through performance and arts: health education in Cambodian forest villages	Cambodia	Mothers and children	Live	2021
Neglected diseases	Williams et al. (57)	Shadow Puppets and Neglected Diseases: A Qualitative Evaluation of a Health Promotion Performance in Rural Indonesia	Indonesia	General population	Live	2018
Tuberculosis	Thomas et al. (58)	Effectiveness of TB sensitization initiatives in improving the involvement of self help group members in rural TB control in south India	India	General population	Live	2016

We performed a rapid assessment of more recent literature in PubMed from 2006 to 2021 systematically searching studies using the terms “Music AND Health AND Intervention” extracted from all fields of papers, and studies for specific diseases [i.e., (“Music OR Song^*^”) AND (“HIV” OR “Ebola” OR “Covid” OR “Malaria”)] extracted from papers' title and abstract. From a total of 4,028 studies found, we retrieved 37 studies specifically describing a music intervention campaign related to infectious diseases, excluding 3,987 that were not related to preventing an infectious agent and four reviews of the literature (included in the main text but not in the table). The “Live” category on Delivery media includes live music performances, classroom activities, local actions in the field, and focus groups.

Most music interventions have focused on increasing awareness of individual risky behaviors (59). For example, hip hop music was used to explain that HIV can be transmitted through unprotected sex and drug use (36, 39). Recent music campaigns have clarified that COVID-19 is an airborne transmitted disease (30). Campaigns focusing on preventive behaviors such as hand washing have targeted both COVID-19 (27, 32) and Ebola (60). Likewise, the appropriate use of bed nets was central to malaria prevention campaigns (55). Music interventions have also aimed at limiting social risky behaviors such as contact with corpses during burials during an Ebola outbreak or physical proximity during the COVID-19 pandemic (28, 61). Finally, music campaigns have been advocated to reduce COVID-19 vaccine hesitancy (29).

Several of these examples provided evidence of the use of music in increasing knowledge and attitudes toward disease prevention [see reviews by Robinson et al. (59), Bunn et al. (20), and Sonke et al. (62)]. For example, music interventions increased knowledge of HIV/AIDS risk factors among adolescents of New York City (25, 36, 43). However, there is limited scientific data demonstrating the impact of music in reducing disease burden at the population level (63).

## Essential Steps for a Health Music Intervention

Based on examples of effective health campaigns (64–68) and on our own experience, we propose that successfully developing and delivering musical messages to prevent EIDs requires at least four steps: (1) Establishing a task force or working group; (2) Identifying the most critical preventative measures for inclusion in the composition of a “catchy” song that is understandable and relates to the target audience; (3) Delivering the song to identified target audiences with sufficient intensity/frequency; and (4) Monitoring and evaluating the effectiveness of the music campaign.

### Step 1: “That's What Friends Are For”

Establishing a task force promptly, including musicians and members of the local community, can critically contribute to the success of an intervention. Failure to do so will likely limit the delivery of the preventive messages, even if these are based on scientific evidence (e.g., hand washing). The inclusion of scientists representing a range of disciplines is beneficial (20, 69, 70). However, in practice, cultural divergences and mismatched expectations may create barriers. Misunderstandings often arise over financial matters in that economic precarity on the part of musicians may compromise their involvement, while scientists might struggle to obtain funding matching musicians' expectations. The motivation to deliver a musically-sound message to help society might outweigh many obstacles, because many musicians are conscious of their important role in contributing to overall societal wellbeing [e.g., rappers and griots in Africa (71)] and, to their local community (Box 1). In fact, several initiatives related to disease prevention emanate from the musicians themselves (e.g., MC Fioti's COVID-19 “vaccine anthem” in Brazil). Collaborations with scientists during these initiatives can refine evidence-based messages and test the effectiveness of campaigns.

Box 1 Mozambican singer-songwriter Feliciano dos Santos has used traditional music to promote health messages and mobilize communities in impoverished rural areas of Mozambique since the early 1990s. His songs raise awareness about major health issues driven by poor sanitation and access to clean water (e.g., “Wash your hands” song by Massukos, Supplementary Table 1). He has chosen music because, in Africa, it is central to cultural expression and identity, and provides many forms of communication. He works closely with communities to ensure that his songs are relevant to the problems they need to solve and that the communities themselves develop a sense of ownership of these messages. This way of working enables him to create a bond with the direct beneficiaries and to learn about local rhythms and songs, which in turn influence and transform his own music. His work with communities goes hand in hand with engagement of government partners. This is essential to influence policies around healthcare delivery and access key information to evaluate the effectiveness of his music-inspired approaches. Like in communities, ownership is central also at this level so to motivate meaningful action and effect sustainable change. Picture: Feliciano dos Santos and his band standing on the back of a track to promote disease prevention in a rural village of Mozambique.

**Figure:**
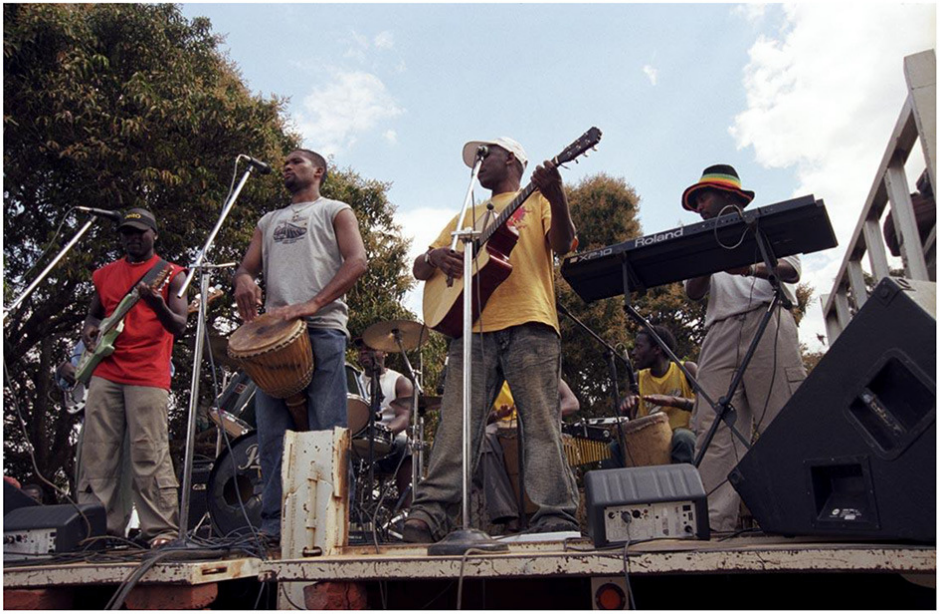

### Step 2: “You Can't Always get What You Want”

For many infectious diseases, preventive measures to reduce transmission risks are well-known, including hand washing, social distancing, using personal protective equipment, and sanitation (6, 67). In such cases, selecting behaviors to be promoted in songs is relatively straightforward. However, when EIDs emerge, preventive measures are not necessarily well-known. Thus, the task force will have to balance the need for early message broadcasting with that of gathering sufficient scientific evidence to convey the appropriate message. As seen during the COVID-19 pandemic, an accurate understanding of the most relevant preventive behaviors and interventions (e.g., use of masks, hand washing, and vaccination) requires time and messages might need changing iteratively depending on the epidemiological situation. In addition, it might not be possible to convey multiple messages in a single song, to avoid both confusion and lack of message retention (66). Thus, we recommend selecting preventive messages during song composition and adapting campaigns to changing epidemiological patterns. This might require the development of multiple songs over time or tailored to populations with different levels of EID risk. Finally, there is a risk of conveying a message that is misinterpreted by the population leading to unintended consequences, such as negative attitudes about infected individuals or suspect wildlife reservoirs. These risks should be considered during an open dialogue between social scientists, the local community, and public health authorities, especially given the limited time for extensive piloting in crisis situations. Overall, balancing possible risks and benefits might limit the amount and type of information delivered in each song.

### Step 3: “How Do You Keep the Music Playing”

Effective mass media campaigns typically require repeat broadcasting of preventive messages through the radio or television. For example, previous mass health media campaigns have suggested using a “message saturation” strategy for radio broadcasting to penetrate the audience, with 6–12 messages per day over several months (72, 73). The type of funding necessary to attain such coverage is often unavailable at the start of an outbreak, particularly when an EID affects a low-income country (e.g., Ebola outbreaks). Early engagement of national and international funders and the media industry is therefore essential to obtain the funds required to achieve the desired broadcasting frequency and population exposure. Wide broadcasting of health awareness messages becomes even more critical when EIDs spread over large geographic areas and have the potential to become a pandemic. Additional challenges arise from adapting songs to further cultural backgrounds, languages, and populations (e.g., from rural to urban areas). Furthermore, society often responds to both musical messages and to performers. Thus, engaging appropriate messengers or influencers with sufficient notoriety to engage a large population and promote desired behaviors is key (68, 74), as demonstrated by the “Natural Opinion leader model” used in HIV prevention (25). However, this further entails securing adequate funding and motivating prominent musicians to promote health messages that might not generate high coverage and interest. Open dialogue with musicians through online round tables, seminars, and direct communication is an essential contributor to such initiatives.

### Step 4: “Show Me the Way”

A key issue of music-based interventions is how to monitor and test their effectiveness in improving preventative practices and reducing disease burden at the population level. This is particularly challenging when dealing with the often-unpredictable nature of EID because data prior to an intervention are often unavailable. Moreover, the need to act rapidly precludes the design and deployment of scientifically sound methods to monitor awareness programs as they are implemented. The need for a rapid response can also make the use of “control groups” (that do not receive the intervention) unethical. Disentangling the specific effect of musical campaigns on reducing population-level disease burden is challenging, since music is often used in addition to other preventive measures (e.g., distribution of soap or vaccination campaigns). Indeed, the role of music campaigns is typically to improve the uptake or adherence to these other measures, and therefore will very much depend on their availability, accessibility, and appropriateness. Knowledge alone will not necessarily translate into improved practices for many reasons that need to be better understood.

Despite these challenges, several promising initiatives are attempting to test the effectiveness of art-based campaigns in reducing disease burden. Methods include the use of quantitative disease incidence data (63) and of approaches to obtain qualitative data through surveys/interviews (55) that could be adapted to the specific challenges of EIDs. Work led by the Development Media International (https://www.developmentmedia.net/) has focused on evaluating mass media campaigns delivered by radio, television, and mobile videos using cluster randomized controlled trials (RCTs). In this design, a randomized group of subjects (cluster) is used for the evaluation, which enables large-scale assessments. For example, Sarrassat et al. (63) used data from health facilities and from cross-sectional household surveys conducted before, during, and after radio campaigns to evaluate whether they could reduce under-five child mortality in Burkina Faso. Similarly, Lemieux et al. (25) used questionnaires to evaluate a more localized music-based HIV prevention intervention among urban adolescents of three high schools in the USA.

## “Ebola Is Back” by the “Gondwana Ebola Task Force”: A Rapid Response to an EID Outbreak Based on Musicians' Solidarity

In this section, we describe our experience in implementing music-based interventions in response to the 2021 Ebola outbreak (75) using the above four-step framework.

### Step 1

Aiming to avoid the delayed community awareness and devastating consequences of the previous outbreak in Guinea and neighboring countries (6), we created the “Ebola Gondwana task force” the day a new outbreak was officially announced [14^th^ of February (75)]. The name “Gondwana” is derived from the supercontinental block that included both Africa and Latin America during the Proterozoic era, reflecting the inclusion of both Latin American and African musicians—from Chile, Brazil, Liberia, and Guinea—and scientists—from Chile and Cameroon. The mandate of the task force was to work with local communities to create awareness about the outbreak as quickly as possible through musical content that would translate science-based prevention guidelines into more accessible messages developed by community members and local musicians.

Due to the COVID-19 pandemic, all communication was done virtually. Our task force immediately contacted several stakeholders responsible for handling the outbreak, including the Regional Office for Africa of the World Health Organization (WHO), the Liberia Ministry of Health, and the United Nations Children's Emergency Fund (UNICEF) in Guinea to make them aware of our initiative and keep them posted on the development of our musical material. Since a member of our group (P.T.D) was based in the city of Zor Zor in Liberia, 65 km east from the first case declared in Nzerekore-Guinea, we hired one local assistant in Liberia and Guinea who contacted local musicians and members of the community to co-develop preventive messages, discuss airtime with local radios, and continuously monitor the epidemiological situation.

### Step 2

We first created preventive messages based on the WHO's guidelines for Ebola prevention as well as lessons from previous outbreaks. This promoted hand washing with soap, avoiding contact with corpses during funerals and visiting a doctor if people develop symptoms indicative of Ebola disease (76). The message content was discussed with an anthropologist working in the area, members of the local community and other musicians working in Africa (F.D.S). In Liberia, our local assistant engaged with two community members (female and male) recording 3–4 min Ebola preventive messages in Loma language (spoken in Liberia and Guinea). Two Latin American musicians (Skyfonik from Chile and Choro de Favela from Brazil) of the task force then added African-style background music to the lyrics. In Guinea, our local assistant in Nzerekore recorded three women giving 1-min prevention messages in three languages (Kpelle, Mania, and Loma) to which we added a musical beat.

A member of our team (J.A.B) composed the lyrics and chords of the “Ebola is back” song in French. This was subsequently arranged, and studio recorded in Nzerekore by the locally known group “Combattant compétent au mic” comprising three musicians (Competent, La Kaka, and Charly). This group was supported by a collective of musicians that contributed to the song in five local languages. The song included key Ebola and COVID-19 prevention messages such as “Ebola is back but we can kick it out, let's wash our hands,” “Ebola is back so we shouldn't drag our feet, let's not touch the dead” or “Let's wear masks, night and day,” and “Ebola and your friend COVID, this is our home, the vaccine will kick you out.”

### Step 3

In Liberia, we paid the community-radio station in Zor Zor (Radio Life) to broadcast these messages for a 2-week period twice a day (morning and afternoon) from February 18th. The radio also broadcast the message at other times of the day, hosted live discussions with the community on Ebola and continued broadcasting the message after the initial 2 weeks. In Guinea, we paid one of Nzerekore's largest radio stations, “Radio Espace Forêt,” to broadcast the 1-min preventive messages once a day for 2 weeks from February 25^th^. We also shared the message with UNICEF in Nzerekore. The “Ebola is back” song was broadcast on two main radio stations in Nzerekore particularly suited to reach rural and isolated communities: “Radio Djoma FM” (broadcast free of charge from March 17^th^ and including a live interview with the musicians) and “Emergence FM” (2-week broadcasting twice a day from April 17^th^). Finally, the song was made freely available online (https://soundcloud.com/unabenradio/ebola-volvio-ebola-est-de-retour) so that it could be shared with NGOs and researchers. The campaign was also posted in our research group's social media (@themonkey_lab). No further action was undertaken until Guinea declared that the outbreak was over on the 19th of June 2021 (76). The project cost 1,200 dollars, raised in Chile through contributions by family and friends. Funds were transferred directly to local assistants and musicians to cover related field activities, studio recording, and musician time. Half of the money was needed to pay for radio airtime. All Latin American musicians and scientists contributed free of charge. The enthusiasm and daily communication between researchers and musicians largely contributed to the rapid and successful broadcasting. However, no funds were available to broadcast the messages for a longer period or at higher daily frequency.

### Step 4

Given the urgency of the response and our limited funding to hire local assistants for long periods of time, we were unable to monitor the effectiveness of this campaign in terms of adoption of individual and social preventive behaviors. Similar limitations likely apply to other outbreak situations, which could help explaining the limited information available on the effectiveness of these music interventions.

## Conclusion: “The Final Countdown”

The COVID-19 pandemic illustrates the impacts of EID spread in a globalized world, highlighting how innovative approaches are needed to engage communities in adopting and sustaining behaviors to prevent disease transmission. Music has great potential to deliver messages to reduce EID risk, but knowledge gaps remain regarding the effectiveness and reach of music-based health campaigns. For example, very few studies have evaluated whether music interventions reduce risky behaviors and subsequent disease burden. In the case of fast-moving EIDs, teasing apart the contribution of different preventative measures (or factors that influence their uptake) can be difficult and would require appropriate statistical designs and/or modeling counterfactuals. The intrinsic unpredictability of EID outbreaks is also a major obstacle to securing funding to develop and widely broadcast music-based preventive messages at an early stage and to evaluate their impacts. We therefore recommend that long-term collaborations are created between musicians, scientists, government media departments, and international agencies dedicated to disease prevention. Collaborating with public health authorities can contribute to disseminating messages to a wide audience and can make disease data accessible to evaluate these music interventions.

## Author Contributions

JAB, KH, and TL conceived and designed the study. JAB, CC, and RMS analyzed the data. JAB, CC, TL, and RMS drafted the paper. JAB, CC, TL, KH, RMS, PTD, and FDS edited the paper. All authors read, commented, and approved the final manuscript. All authors contributed to the article and approved the submitted version.

## Funding

JAB was funded by the National Fund for the Scientific and Technological of Chile (FONDECYT-Iniciación, grant number 11181017). RMS and KH are funded by a Wellcome Trust grant (207569/Z/17/Z). The funders had no role in study design, data collection and analysis, decision to publish, or preparation of the manuscript.

## Conflict of Interest

The authors declare that the research was conducted in the absence of any commercial or financial relationships that could be construed as a potential conflict of interest.

## Publisher's Note

All claims expressed in this article are solely those of the authors and do not necessarily represent those of their affiliated organizations, or those of the publisher, the editors and the reviewers. Any product that may be evaluated in this article, or claim that may be made by its manufacturer, is not guaranteed or endorsed by the publisher.
