# Supplementary material for: The Power of Music to Prevent and Control Emerging Infectious Diseases
Source: Front Med (Lausanne). 2021 Nov 25;8:756152. doi: 10.3389/fmed.2021.756152 (PMC8655130; doi:10.3389/fmed.2021.756152)
Supplement: Supplementary file 1 [file Table_1.docx]

**Table S1. Examples of music interventions to prevent and control endemic and emerging infectious diseases found on YouTube.**

Songs were selected from a non-exhaustive search in YouTube using keywords ‘Music’ and ‘disease’ or ‘health intervention’ or ‘COVID’ or ‘Ebola’ or ‘disease prevention campaign’. Search was sorted by number of views and videos were fully watched to ensure the content related to infectious disease prevention.

| **Year** | **Country** | **Target population** | **Disease** | **Song Name** | **Agency or musical group** | **Link** |
| --- | --- | --- | --- | --- | --- | --- |
| 1996 | United States | General population | HIV | America is Dying Slowly | Wu Tang Clan | https://www.youtube.com/watch?v=q-fnXIHv8-Y |
| 2010 | USA | Children | Infectious diseases | Happy Handwashing Song | Centers for Disease Control and Prevention | https://www.youtube.com/watch?v=kHPQrYthn6M |
| 2012 | Mozambique | General population | General preventive measures | Wash Your Hands (Tissambe Manja) | Massukos | https://www.youtube.com/watch?v=QbpGjWYnfrY |
| 2014 | United Kingdom | General population | Ebola | Do they Know It’s Christmas | Band Aid 30 | https://www.youtube.com/watch?v=-w7jyVHocTk |
| 2014 | Liberia | General population | Ebola | Ebola is real | The Talented Young Brothers | <https://www.youtube.com/watch?v=BUpizQ7FdFA> |
| 2014 | Liberia | General population | Ebola | Ebola in town | Shadow | <https://www.youtube.com/watch?v=XGltVAJ4JCk> |
| 2014 | West Africa | General population | Ebola | Ebola- We can overcome | Different artists from Africa and USA | <https://www.youtube.com/watch?v=ADWxlccX2Nw> |
| 2014 | Liberia | General population | Ebola | Ebola – Outbreak in West Africa | Black Diamond | <https://www.youtube.com/watch?v=SLan7OZnT_w> |
| 2014 | Liberia | General population | Ebola | Ebola Hope Song | Lib All Star | <https://www.youtube.com/watch?v=cFwNZ_7GLcg> |
| 2014 | Liberia | General population | Ebola | Ebola | G-Nice, T-Boy and Living Stone | <https://www.youtube.com/watch?v=9QWO-FfYEfc> |
| 2015 | Sierra Leone | General population | Ebola | Bye Bye Ebola | Block Jones ft Freetown Uncut | <https://www.youtube.com/watch?v=YRhXoLjHruM> |
| 2015 | Haiti | Youth | General preventive measures | Let’s wash our hands | GOALS | <https://www.youtube.com/watch?v=fmvT9lVKFZ4> |
| 2016 | Australia | Indigenous children | Skin, throat and ears infections | Merredin “Gotta Keep it Strong” | Indigenous Hip Hop Projects | <https://www.youtube.com/watch?v=bhLU5Qnf0n8> |
| 2017 | Ecuador | General population | COVID-19 | La Cumbia Del Coronavirus | General Hospital Ambato | https://www.[youtube.com/watch?v=xlvID2tk8N8](http://youtube.com/watch?v=xlvID2tk8N8) |
| 2018 | Singapore | General population | Infectious diseases | Let's F.I.G.H.T. The Spread of Infectious Diseases | Singapore’s Health Promotion Board (HPB) | https://www.youtube.com/watch?v=iihg76chgPU |
| 2020 | Spain | General population | COVID-19 | Stay Homa (Confination Song II) | Stay Homas ft Mr Wilson | <https://www.youtube.com/watch?v=mkJXh52fF8c> |
| 2020 | Vietnam | General population | COVID-19 | Jealous Coronavirus  (Ghen Cô Vy) | Khắc Hưng/ Vietnam National Institute of Occupational and Environmental Health (NIOEH) | https://www.youtube.com/watch?v=BtulL3oArQw |
| 2020 | England | General population | COVID-19 | Spreadin’(Coronavirus) | Psychs | https://www.youtube.com/watch?v=Zg2dmvvew_o&t=81s |
| 2020 | Vietnam | General population | COVID-19 | Let’s Fight COVID (VIỆT NAM ƠI! ĐÁNH BAY COVID) | Minh Beta | https://www.youtube.com/watch?v=tSiK7U46PfA |
| 2020 | Canada | General population | COVID-19 | Coronavirus | Tom MacDonald | https://www.youtube.com/watch?v=QPhe2n-p-mw |
| 2021 | Brasil | General population | COVID-19 | Bum Bum Tam Tam | MC Fioti | https://www.youtube.com/watch?v=yQ8xJHuW7TY |
| 2021 | France | General population | COVID-19 | Je me souviens | Mcfly e Carlito | https://www.youtube.com/watch?v=t4h8j9xLyxQ |
